# Bilingualism Accentuates Children's Conversational Understanding

**DOI:** 10.1371/journal.pone.0009004

**Published:** 2010-02-03

**Authors:** Michael Siegal, Luca Surian, Ayumi Matsuo, Alessandra Geraci, Laura Iozzi, Yuko Okumura, Shoji Itakura

**Affiliations:** 1 Department of Psychology, University of Sheffield, Sheffield, United Kingdom; 2 Department of Psychology, University of Trieste, Trieste, Italy; 3 Department of Cognitive Sciences and Center for Mind/Brain Studies (CIMeC), University of Trento, Rovereto, Italy; 4 School of English Literature, Language and Linguistics, University of Sheffield, Sheffield, United Kingdom; 5 Department of Psychology, Kyoto University, Kyoto, Japan; Victoria University of Wellington, New Zealand

## Abstract

**Background:**

Although bilingualism is prevalent throughout the world, little is known about the extent to which it influences children's conversational understanding. Our investigation involved children aged 3–6 years exposed to one or more of four major languages: English, German, Italian, and Japanese. In two experiments, we examined the children's ability to identify responses to questions as violations of conversational maxims (to be informative and avoid redundancy, to speak the truth, be relevant, and be polite).

**Principal Findings:**

In Experiment 1, with increasing age, children showed greater sensitivity to maxim violations. Children in Italy who were bilingual in German and Italian (with German as the dominant language L1) significantly outperformed Italian monolinguals. In Experiment 2, children in England who were bilingual in English and Japanese (with English as L1) significantly outperformed Japanese monolinguals in Japan with vocabulary age partialled out.

**Conclusions:**

As the monolingual and bilingual groups had a similar family SES background (Experiment 1) and similar family cultural identity (Experiment 2), these results point to a specific role for early bilingualism in accentuating children's developing ability to appreciate effective communicative responses.

## Introduction

Bilingualism is present to some extent in every society and at least half of the world's population uses more than one language in everyday life. From this perspective, it is monolingualism rather than bilingualism that is uncommon [1], [2]. Yet the developmental consequences of early childhood bilingualism remain controversial [3], [4]. In Britain, for example, misgivings about its importance have resulted in decreasing numbers of children from English-speaking homes studying a second language [5]. Here we report evidence that early access to a second language promotes young children's awareness of effective responses in conversation with others.

Bilingualism has been found to have a positive effect on children's ability to judge grammar and to substitute symbols [6], [7]. In this sense, exposure to more than one language appears to facilitate children's metalinguistic awareness. There is also evidence, albeit inconsistent, that bilingualism advantages attentional and executive control processes [e.g., 8]. Moreover, research on conversational interactions has shown that, from an early age, bilingual children can make appropriate choices of the language for communication and can differentiate their language use in ways that are sensitive to context [9]–[13]. Findings of flexibility in the representation and usage of language and enhanced executive control indicate that early bilingualism should be accompanied by advanced skills in identifying effective responses in conversation. However, little is known about the extent to which bilingualism influences performance on measures of conversational understanding–a process that is often central to cognitive development and learning [14], [15].

In his widely influential analysis, the philosopher Paul Grice [16] depicted communication as a cooperative exchange. He proposed that appreciation of certain conversational maxims provides the foundation for pragmatic competence. These maxims enjoin speakers to “say no more or no less than is required for the purpose of the (talk) exchange” (Maxims of *Quantity*), “tell the truth and avoid statements for which there is insufficient evidence (Maxims of *Quality*)”, “be relevant (Maxim of *Relation*)”, and “avoid ambiguity, confusion and obscurity (Maxims of *Manner*).” To characterize the nature of effective communication more fully, Grice also discussed the need to invoke other maxims such as “be polite” (Maxim of *Politeness*) that have traditionally been recognized as key to conversational processes [17]–[21].

**Figure 1:**
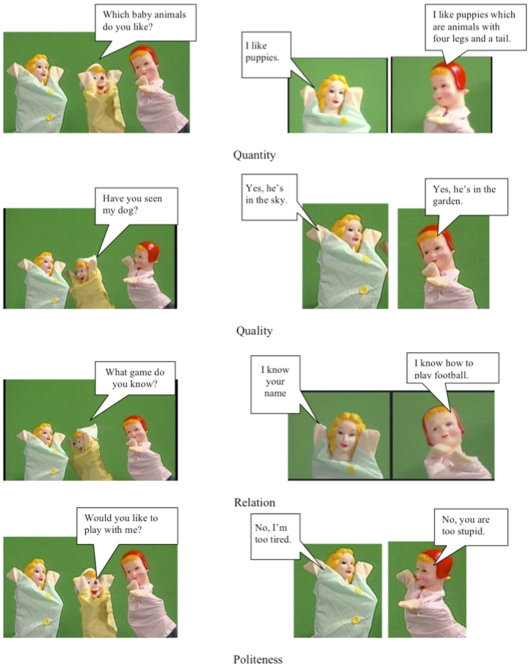
Examples of items in the Quantity, Quality, Relation, and Politeness maxim components of the Conversational Violations Test.

Even in the earliest years, children demonstrate sensitivity to conversational maxims [22], [23]. Given studies suggesting that bilingualism serves to promote children's metalinguistic awareness, the aim of a recent investigation [24] was to determine whether bilingual children aged 3 to 6 years excel in their recognition of certain key instances of maxim violations compared to their monolingual counterparts. For this purpose, a Conversational Violations Test (CVT) was employed to examine children's ability to identify utterances that violate the Maxims of Quantity, Quality, Relation, and Politeness. Previous studies have shown that typically developing children are advantaged on the CVT compared to children with limited access to conversation such as children with autism and deaf children with hearing parents [25], [26]. To compare the performance of monolinguals and bilinguals, the CVT was given to two groups of children from the Trieste, Italy, and the Slovenian border area: one that was monolingual in Italian and the other bilingual in Slovenian (L1) and Italian (L2) Using a laptop computer, children were shown a DVD in which short conversational exchanges in Italian were staged by three doll speakers, one male and two female. For each episode, one of the two female speakers asked a question to the other two speakers who each gave a short answer. One answer violated a conversational maxim and the other did not (Figure 1). The children were asked to “point to the doll that said something silly or rude.” Though comparatively delayed in their L2 as shown by performance on picture vocabulary tests, children who were bilingual in Italian and Slovenian (with Slovenian as the dominant language L1 spoken at home) generally outperformed those who were either monolingual in Italian or Slovenian in detecting utterances that violate conversational maxims with older children outperforming younger ones.

Contrary to the view that an early bilingual advantage is based on parental interpersonal sensitivity rather than enhanced access to language, it has long been observed that parents' motivation in sending their children to second language schools is to secure better employment and social conditions for their children rather than by a perceived need to engage in dialogues with speakers of another language [27]. Nevertheless, these initial results were restricted to children with proficiency in either Italian or Slovenian or both languages. There was no comparison of bilinguals' CVT performance in both their languages and no direct measure of socioeconomic status despite evidence that differences between bilingual and monolingual children on measures of cognitive development may reflect non-linguistic factors based on pre-existing SES differences [28], [29] and the contentious debate over whether such factors may overshadow a bilingual advantage [30], [31].

To examine these issues, the present research involved children aged 3 to 6 years exposed to one or more of four major languages: English, German, Italian, and Japanese. All children participated with informed parental consent. In Experiment 1, we compared performance on an Italian version of the CVT by children bilingual in German and Italian (with German as L1 and Italian as L2) with Italian monolingual children. In Experiment 2, we sought to compare performance on the CVT in two other language groups: children bilingual in English and Japanese (with English as L1 and Japanese as L2) with Japanese monolingual children. The bilingual group received the CVT in both English and Japanese permitting a cross-language comparison. In our comparison of these two groups, children received a measure of verbal mental age. We also sought to provide innovative evidence on possible cultural differences between the language groups by questioning mothers on their Japanese identity. Moreover, as food and eating contribute importantly to communicative expectations and socialization practices [32], [33], we examined mothers' food preferences.

For both experiments, we predicted that, with increasing age, children would significantly improve in the ability to detect maxim violations and that bilingual children would outperform their monolingual counterparts.

## Methods

This research was approved by the ethical review board of the EU Sixth Framework and the relevant ethical review committees of the University of Trieste, the University of Trento, and Kyoto University. All children participated with written informed parental consent.

### Experiment 1

#### Participants

These were 36 German-Italian bilingual children and 41 Italian monolingual children attending Italian preschools in Bolzano in the Trentino-Alto Adige region of Italy near the Austrian border where standard German is spoken, albeit with a distinctive regional accent. As in previous research [24], the children were divided into two age groups ranging from 37 to 55 months and from 56 to 75 months. The mean ages of the 18 younger bilingual and 22 monolingual children were 46.8 months (SD  = 5.4) and 45.6 months (SD  = 4.9) respectively. The mean ages of the 18 bilingual and 19 monolingual children in the older group were 66.6 months (SD  = 5.7) and 63.6 months (SD  = 5.2) respectively. All children had been enrolled in preschool from the age of 3 years. Both parents of the monolinguals used Italian at home. In the bilingual group, at least one parent used German and the children had a predominantly German home language environment, although all the bilingual children were exposed to both German and Italian from birth. Mean years of education for mothers and fathers of bilingual children were 12.10 (SD  = 2.84) and 11.64 (SD  = 3.04) respectively. Comparable figures for mothers and fathers of monolingual children were 12.63 (SD  = 3.28) and 13.00 (SD  = 3.00) A 2 (parents) X 2 (age groups) X 2 (language groups) analysis of variance on years of education yielded no significant main or interaction effects, F's(1,73) ≤2.17, p's≥0.15, η^2^≤0.029.

#### Procedure

The children in the two groups were given the CVT in Italian by a native Italian speaker. Alpha reliability was .64 (*N* = 77). Small changes were made in the content of the CVT items to reflect cultural familiarity. For example, in one of the items on Quantity in the Italian version, the puppet response “milk with biscuits” was substituted for “cornflakes and then a boiled egg and toast” in the English version.

In previous studies [26], [34], the CVT consisted of 25 items rather than the 20 items–five for each maxim–used in the present investigation. The additional five items were intended to examine performance on a sub-maxim of Quantity concerning the need to avoid saying too little for effective communication. These items were omitted in our studies as recent work [24] has indicated that the context of such items may be perceived as ambiguous.

As a measure of attention and inhibition, the children were also given the Day-Night task [for details], [see 24,35]. On this measure, children are shown pictures of a sun and moon and are required to respond *day* when they see a moon picture and *night* when they see a sun picture.

### Experiment 2

#### Participants

These were 33 English-Japanese bilingual children and 59 Japanese monolingual children ranging from 55 to 85 months. The mean ages of the bilingual and monolingual children were 68 months (SD  = 8.3) and 67 months (SD  = 8.6) respectively and were comparable in age to the older group of children tested in Experiment 1 who demonstrated significant bilingualism effects on the CVT. The bilingual children were from Derby, Leeds, Manchester, and Sheffield in England. At least one of their parents was Japanese and a mixture of English and Japanese was used in their home language environment. Although some children had a predominantly Japanese home language environment, all children were exposed to both languages from birth or before the age of 2 years. The children attended English language schools and also a Saturday school and playgroups where Japanese was used and where they were tested in a quiet room. The monolingual children were from Kyoto, Japan, and attended Japanese language schools. Some of the children were tested in a quiet room in their school; others were tested in a university child development laboratory. Both parents of the monolinguals used Japanese at home.

#### Procedure

A bilingual experimenter tested the bilingual children in two sessions separated by a 1–2 week interval. Half the children received testing in English first, including the CVT in English, and in Japanese second, including the CVT in Japanese. The order was reversed for the other children. A Japanese native speaker tested the monolingual children in Japanese. Small changes were made in the content of the CVT items for the two versions to reflect cultural familiarity. For example, in one of the items on Relation in the Japanese version given to the monolinguals, the puppet response “I know how to play baseball” was substituted for “I know how to play football” in the English version. Alpha reliability was 0.54 for the English CVT (N  = 33) and 0.75 for the Japanese CVT (N = 92)

Both the bilingual and monolingual children were given the Day-Night task in Japanese. As a measure of vocabulary mental age (VMA), both groups were also given the Japanese Picture Vocabulary Test [36]. As well, the bilingual children also received the British Picture Vocabulary Scale [37]. The bilinguals' VMA scores in English (M = 69 months, SD  = 13.8) were significantly higher than their scores in Japanese (M = 57 months, SD  = 11.1), F(1,64) = 17.39, p<0.0001, η^2^ = .214, with 28 of the 33 children having a higher English than Japanese VMA. In addition, in keeping with the bilingual children are often delayed in their vocabulary comprehension in individual languages though not necessarily in overall vocabulary size [38], the bilinguals' Japanese VMA scores were significantly lower than those of the monolingual Japanese children (M = 75 months, SD  = 14.84), F(1,90) = 38.30, p<0.0001, η^2^ = .299. Moreover, their English VMA scores also tended to be lower than the VMA scores of the monolinguals in Japanese, F(1,90) = 3.07, p<0.09, η^2^ = .033. There were no significant order effects on the bilinguals' VMA scores in either language.

As a means toward determining whether the children in both countries could be considered to be similar in family cultural identity and food preferences, a brief questionnaire was administered in Japanese to a sub-sample of mothers: 17 mothers of the monolinguals in Japan (age range, 32–47 years, M = 37.0, SD  = 3.8) and 19 mothers of the bilinguals in England (age range, 32–47 years, M = 38.5, SD  = 3.5) Following a procedure carried out previously with a sample of Australian adults [39], the mothers were asked to indicate their sense of a Japanese identity in a yes or no response to the items, “I think being Japanese is one of the most important things about me” and “I definitely want to have Japan as my permanent main home.” On a scale from 1 (highly unfavorable) to 9 (high favorable), they were also asked to rate 7 western/international foods that are common in Japan (apples, black coffee, brocolli, carrots, chocolate, hamburger, and milk) and 7 Asian/Japanese foods (curry rice, furikake, karashimentai, natto, shiojake, tofu, and umebosi) The mothers of the bilingual children were given the questionnaire at one of the testing sites in England. The mothers of the monolingual children completed the questionnaire in a university laboratory setting while their children were tested. None of the mothers of children in either group refused to complete the questionnaire.

## Results

### Experiment 1

**Figure 2:**
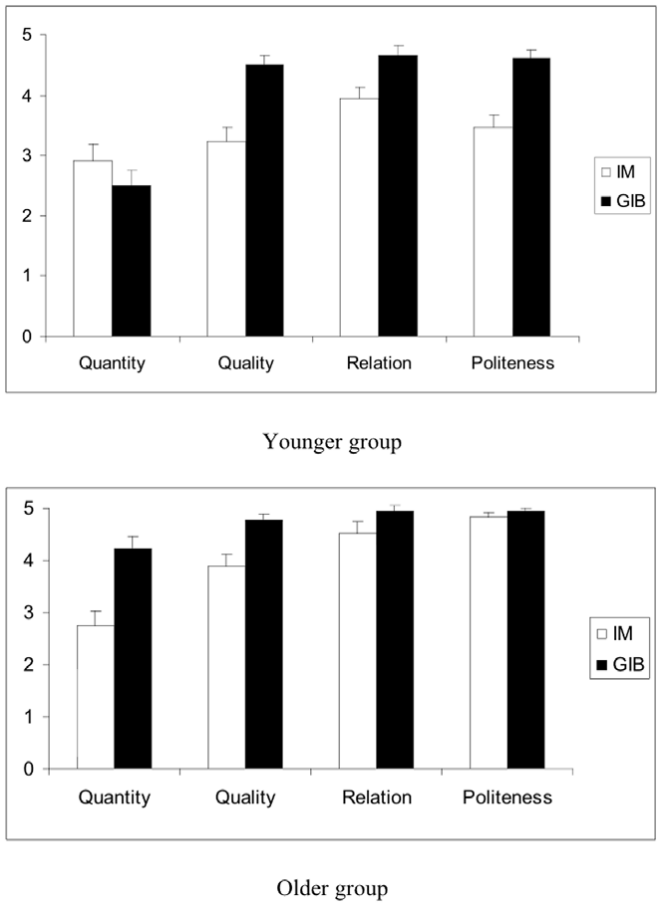
Mean CVT maxim scores (out of 5) in Experiment 1for the Italian monolinguals (IM) and German-Italian bilinguals (GIB) in the younger (37 to 55 months) and older (56 to 75 months) age groups.

A 2 (age groups) X 2 (language groups) X 4 (maxims) analysis of variance on CVT scores showed significant main effects for age group, F(1,73) = 41.29, p<0.0001, η^2^ = 0.361, and for language group, F(1,73) = 50.85, p<0.0001, η^2^ = 0.411. As predicted, the bilingual children outperformed their monolingual counterparts with a mean score of 17.58 (SD  = 1.83) out of 20 compared to 14.68 (SD  = 2.36) for the monolinguals. The maxim main effect was also significant, F(3,219) = 44.75, p<0.0001, η^2^ = 0.380. In addition, there was a significant age group X language group X maxim interaction effect, F(3,219) = 10.49, p<0.0001, η^2^ = 0.126 (see Figure 2) The younger bilingual children outperformed their monolingual counterparts on Quality, Relation, and Politeness, t's (38)≥3.02, p's<.005. Comparable differences between the older bilingual and monolingual children were significant for Quality, Quantity, and Relation, t's (35)≥2.42, p's≤.021. Whereas older bilinguals outscored younger bilinguals only on the Quantity Maxim, t (34) = 5.05, p <.0001, older monolinguals outscored younger monolinguals on both Relation, t (39) = 2.34, p <.01, and Politeness, t (39) = 5.43, p <.0001. Further analyses indicated that the younger and older bilingual children and the older monolingual children all scored significantly higher on Quality, Relation, and Politeness than on Quantity, t (17)≥6.18, p's <.0001, t's (18)≥3.08, p's <.0007, and t's (18)≥2.95, p's <.008 respectively. The younger monolingual children scored significantly higher on Relation than on Quantity or Quality, t's (21)≥2.93, p's <.008.

As found previously [24], there were no significant differences between language groups in children's responses on the Day-Night measure. A 2 (age groups) X 2 (language groups) ANOVA on the Day-Night measure revealed a significant age group main effect, F(1,73) = 39.23, p<0.0001, η^2^ = 0.350, and a main effect for language group that fell short of significance, F(1,73) = 3.41, p<0.069, η^2^ = 0.045. The mean score of the bilinguals out of 16 was 14.03 (SD  = 1.25) compared to 14.46 (SD  = 1.55) for the monolinguals. The age group X language group interaction was nonsignificant, F<1.

Years of parental education, seen to be an optimal measure of SES in Italy [40], were quite similar for both groups. Given the link between SES and performance on measures of executive functioning [41], [42], the lack of significant differences between the language groups on the Day-Night task further attests to the comparability of the groups in SES. Therefore the significant CVT difference between the language groups is unlikely to be a function of SES rather than specific to bilingualism itself in terms of an enhanced access to language.

As predicted, in children's developing sensitivity to violations of conversational maxims, bilinguals generally outperformed monolingual children. In both the younger and older age groups, the bilingual advantage was significant on three out of the four maxim components. In the younger children, there was no significant bilingual difference only on Quantity and, in the older children, only on Politeness. In the former case, the younger children's scores on Quantity, whether monolingual or bilingual, lagged significantly behind those on the other three maxims while, in the latter case, the older bilinguals and monolinguals performed equally well on Politeness.

### Experiment 2

Preliminary analyses indicated no significant order of presentation effects on the bilinguals' CVT scores in either English or Japanese. As in Experiment 1, a 2 (language group) X 4 (maxims) analysis of variance on CVT scores in English for the bilingual group and Japanese for the monolingual group yielded a significant main effect for language group, F(1,270) = 4.80, p<0.032, η^2^ = .051. Despite their lower VMA scores, the CVT performance of the children bilingual in English and Japanese with English as L1 (M = 16.57, SD  = 0.98) was significantly higher than those of the Japanese monolinguals (M = 15.30, SD  = 1.04) The maxims main effect was also significant, F(3,270) = 14.15, p<0.0001, η^2^ = 0.136) Children scored significantly higher on the Relation Maxim than on Quantity and Politeness, t's (91)≥2.70, p's <.008, and significantly higher on Quantity and Politeness than on Quality, t's (91)≥3.75, p's <.0001. There were no significant interaction effects. A 2 (language groups) X 4 (maxims) analysis of variance on the Japanese CVT version yielded no significant main or interaction effects.

**Figure 3:**
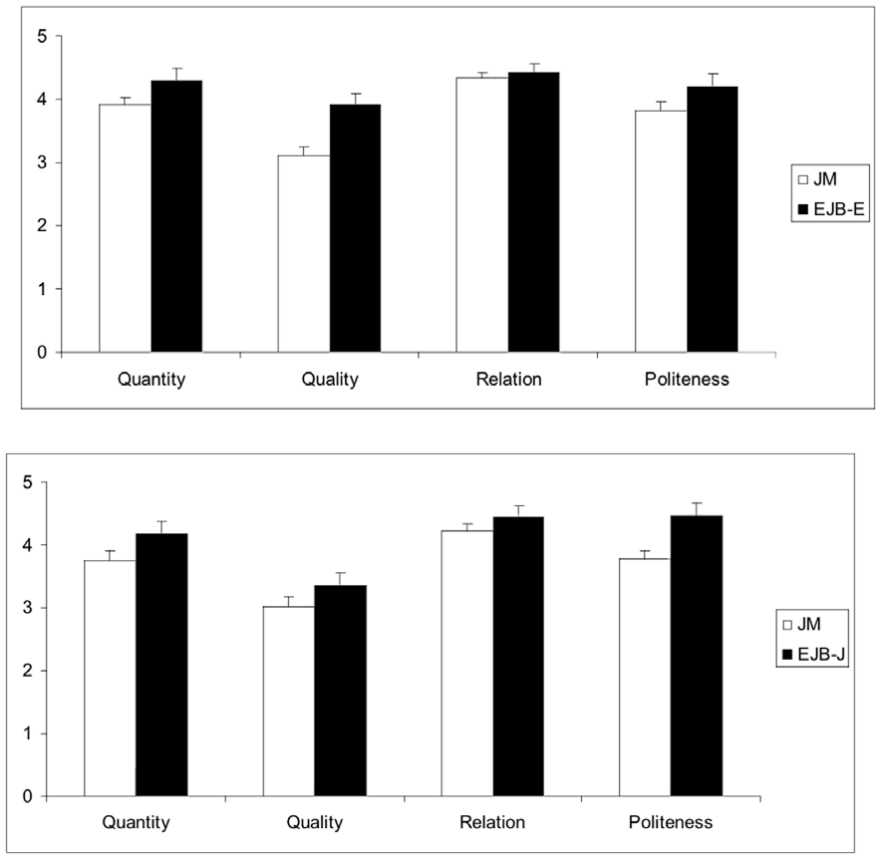
Mean CVT maxim scores (out of 5) in Experiment 2 for the monolinguals in Japanese (JM) and for the English-Japanese bilinguals in English (EJB-E) and Japanese (EJB-J) adjusted for verbal mental age.

Further analyses were carried out to compare CVT performance in the language groups with VMA covaried. The pattern of performance on each maxim is shown in Figure 3. A 2 (language groups) X 4 (maxims) analysis of covariance on CVT scores in English for the bilingual group and Japanese for the monolingual group using English VMA for the bilinguals and Japanese VMA for the monolinguals as a covariate revealed only a significant main effect for language group. As predicted, bilinguals (M_adj_ = 16.82, SD  = 2.14) outperformed monolinguals (M_adj_ = 15.16, SD  = 2.50), F(1,89) = 9.15, p = 0.003, η^2^ = 0.093. Similarly, a 2 (language groups) X 4 (maxims) analysis of covariance using Japanese VMA as a covariate for both groups also revealed only a significant language group main effect, with bilinguals (M_adj_ = 16.45, SD  = 2.77) outperforming monolinguals (M_adj_ = 14.78, SD  = 2.64), F(1,89) = 9.15, p = 0.003, η^2^ = 0.093. F(1,89) = 6.87, p<0.01; η^2^ = 0.072.

The correlation between responses on the English and Japanese CVT versions for the bilingual children was 0.59, p<.001. There were no significant differences between the bilinguals' scores on the Japanese and English versions of the CVT and no significant order of presentation effects. Correlations between VMA and CVT scores in English for the bilingual children and in Japanese for the bilingual and monolingual children were 0.41, 0.51, and 0.37 respectively (all p's<.02, two-tailed). The extent to which children displayed “balanced” or “imbalanced” bilingualism as shown by their VMA in English and Japanese was not associated with their CVT performance. Differences between the bilingual children's scores on the two VMA measures were not significantly correlated with CVT scores in either English or in Japanese (r's<0.11, p's>0.50).

As in Experiment 1, there were no significant differences between the language groups in children's responses on the Day-Night measure, F<1. The mean score of the bilinguals out of 16 was 13.67 (SD  = 4.04) compared to 13.15 (SD  = 3.90) for the monolinguals.

In their questionnaire responses, mothers in both groups overwhelmingly provided responses indicative of a strong Japanese cultural identity. Of the 19 mothers of bilinguals, 14 agreed with the statement, “I think that being Japanese is one of the most important things about me” with 5 disagreeing; 15 mothers agreed with the statement “I definitely want to have Japan as my permanent main home” with 3 unsure and only 2 disagreeing. Of the 17 mothers of monolinguals, 11 agreed with the statement, “I think that being Japanese is one of the most important things about me” with 2 disagreeing and 4 unsure; 13 mothers agreed with the statement “I definitely want to have Japan as my permanent main home” again with 4 unsure. The mothers' ratings for the 14 foods on the questionnaire are shown in Table 1 and are overall quite positive. Unexpectedly, the bilinguals' mothers rated three Japanese foods (karashimentai, natto, shiojake) significantly more favorably than did the mothers of the monolinguals, t's (34)>2.37, p's<.023 two-tailed, whereas the mothers of the monolinguals rated chocolate more favorably than did the bilinguals' mothers, t (34)>2.30, p<.028. There were no significant differences for the other 10 foods.

**Table 1:** Food preferences of mothers of Japanese monolingual children (*N* = 17) in Japan and mothers of English–Japanese bilingual children in England (*N* = 19) rated on a scale from 1 (highly unfavorable) to 9 (highly favorable).

	Monolingual		Bilingual	
Foods	*M*	*SD*	*M*	*SD*
apples	7.41	1.33	7.28	1.97
black coffee	5.29	3.41	5.47	2.62
broccoli	7.18	1.29	6.93	1.90
carrots	6.59	1.50	6.04	1.87
chocolate	8.59	0.87	7.61	1.76
curry rice	7.12	2.00	7.69	2.04
furikake	7.71	1.53	6.57	1.93
hamburger	6.06	3.07	7.3	1.78
karashimentai	6.12	2.03	7.73	1.91
milk	6.71	2.37	5.99	2.07
natto	6.94	1.56	7.79	2.01
shiojake	6.47	2.18	7.72	1.87
tofu	7.71	1.16	7.11	1.95
umebosi	6.94	1.39	7.27	1.88

To examine whether the CVT scores of the children whose mothers responded on the questionnaire differed from those who did not respond, 2 (respondent status) X 2 (language group) X 4 (maxims) ANOVAs were performed on the Japanese CVT scores for both the bilingual and monolingual groups and for the English scores in the bilingual group and the Japanese scores in the monolingual group. In either case, there were no significant main or simple interaction effects differences involving respondent status, F's<1. Similar results emerged from 2 (respondent status) X 2 (language groups) ANOVAs carried out on the Japanese PPVT scores for both the bilingual and monolingual groups and for the English scores in the bilingual group and the Japanese scores in the monolingual group and, F's (1,88) = 1.524, p's>0.20, η^2^ = 0.017.

An issue that arises from our results is whether the bilinguals' superiority on the CVT is similar across language groups. Overall, the CVT scores of the older group of children in Experiment 1 (M = 17.41, SD  = 2.10) were significantly higher than those of similar-aged children in Experiment 2 (M = 15.38, SD  = 2.66), F (1, 127) = 17.08, p<.0001, η^2^ = 0.119. However, as we did not have equivalent VMA scores for the two bilingual and two monolingual groups, differences in performance could be attributed to verbal ability. To address this issue systematically, we compared the older 20 Slovenian-Italian bilinguals and 21 Italian monolingual children [from 24, Experiment 2] who were comparable in age with those of the 33 bilingual English-Japanese and 59 monolingual Japanese children in Experiment 2 of the present investigation. The CVT scores of the four groups (in Italian for the Slovenian-Italian bilingual and Italian monolinguals, in English for the English-Japanese bilinguals, and in Japanese for the Japanese monolinguals) were analyzed in a 2 (language groups: bilingual vs. monolingual) X 2 (cultural groups: Italian vs. Japanese) X 4 (maxims) ANCOVA with VMA scores in the testing language as a covariate. There was a significant language group main effect, F (1, 127) = 33.48, p<.0001, η^2^ = 0.209, indicating that the bilingual children outperformed the monolinguals. Although the cultural group main effect was not significant, F (1,127)<1, there was also a significant language group X cultural group interaction effect, F (1, 127) = 6.68, p = .011, η^2^ = 0.050. The Slovenian-Italian bilingual children (M_adj_ = 4.56, SD  = 0.25) outperformed their English-Japanese counterparts (M_adj_ = 4.14, SD  = 0.57), F(1,51) = 7.94, p  = .007, η^2^ = .135. By contrast, the CVT scores of the monolingual Italians (M_adj_ = 3.86, SD = 0.53) and Japanese (M_adj_ = 3.83, SD  = 0.71) did not differ significantly, F (1,77) = 0.043, p = 0.836, η^2^ = 0.001. In addition, the cultural group X maxim interaction effect was significant, F (3, 381) = 13.36, p<.0001, η^2^ = 0.095. The Japanese-speaking children in Japan and England scored significantly higher on Quantity, Relation, and Politeness than Quality, t's (91)≥22.69, p's <.0001, and significantly higher on Relation than either Quantity or Politeness, t's (91)≥16.15, p<.0001. By contrast, as in Experiment 1, Italian-speaking children in Italy and Slovenia scored significantly higher on Quality, Relation, and Politeness than Quantity, t's (39)≥24.45, p's<.0001, and they also scored significantly higher on Relation than on Quality, t (39) = 5.83, p<.0001. The three-way interaction effect was not significant, F (3,381) = 1.273, p>0.28, η^2^ = 0.010.

## Discussion

The results of our investigation provide support for the position, consistent with evidence that exposure to more than one language facilitates children's metalinguistic awareness, that bilingualism confers an advantage on children's conversational understanding through accentuating their ability to appreciate effective communicative responses. Whether bilingual in German and Italian or English and Japanese, the bilingual advantage in recognizing maxim violations was similar to that reported previously in Slovenian-Italian bilinguals.

In the light of the recent debate on the role of non-linguistic influences in comparisons of monolingual and bilingual children's cognitive task performance [30], [31], our examination of non-linguistic factors in which the two groups may differ seems timely. In Experiment 1, there was no significant difference in years of parental education of the monolingual and bilingual children as an SES measure and, in Experiment 2, the significant bilingual advantage with verbal mental age as a covariate emerged on the CVT whether the bilinguals' scores were in Japanese or English. Moreover, mothers of the English-Japanese bilinguals in England professed at least as strong Japanese cultural identity preferences as mothers of the Japanese monolinguals in Kyoto. Although they at times expressed even more favorable ratings of Asian/Japanese food than did the Kyoto mothers, we believe that owing to regional differences, elsewhere in Japan, such foods would be rated as highly as Japanese living in England. Moreover, mothers in both the bilingual and monolingual groups practice the tradition of preparing traditional *obento* lunches for their children that is important to Japanese socialization practices. Therefore the pattern of a strong Japanese cultural affinity shown by both groups of mothers would appear to rule out family cultural background as an explanation for the CVT advantage shown by the English-Japanese bilinguals in Experiment 2.

Still another possibility is that the bilingual children are exposed to more parental talk about maxims whereas monolingual children learn about maxims from other children. Indeed, bilingual children have been observed to switch languages specifically to gain attention and information from their mothers [12]. On this view, it is not access to more than one language itself that promotes conversational understanding but a specific highlighting of maxims in an adult language different from language used in ordinary discourse with other children that facilitates the advantage shown by bilingual children. We believe that such an interpretation is implausible as, in either Experiments 1 or 2, the communication of the bilingual children with other children was not at all restricted to the language used by adults. Rather, in their exchanges with other children, both languages were often used.

The practice gained by bilingual children at rapidly processing maxim violations and extracting meaning from conversation may free up resources that enable them to close the frequent gap with monolinguals in vocabulary knowledge in individual languages. This process is liable to involve enhanced attention and executive control. Our present investigation was limited in using only one measure of attention and executive control on which no significant differences were found between monolingual and bilingual children. Results in this area have been inconsistent [8], [24], [29], [43]. Additional studies using a wide range of tasks using both behavioral and neuroimaging techniques [e.g., 44] are needed to determine the manner in which bilingual children are advantaged in managing language-specific attentional demands. These measures may involve an examination of the relative “strengths” of bilinguals' languages as shown in a language-switching paradigm in which the task is, for example, to count from 1 to 10 by switching alternatively from one language to another (e.g., counting first in Italian and then in English and back to Italian: uno, two, tre, four, cinque, six, sette, eight, nove, ten) Such measures are internal to the language system and demonstrate the extent to which the return to an L1 is inhibited by the effort needed to shift from processing in an L2 [45]. These may provide a clearer basis for establishing a bilingual advantage in processes of attention and executive control that underpin the development of pragmatics.

Whatever the effects of executive control, the pattern of bilingual advantage in children's conversational understanding is consistent with the position that exposure to more than one language can facilitate performance on key measures of cognitive development such as in the expression of “theory of mind” reasoning in recognizing how holding a false belief can lead to searching for an object in an incorrect location [46], [47]. While bilingual children often confront difficulties in vocabulary comprehension as well as in certain other structural aspects of their L2, our results indicate that they grow increasingly adept with age in identifying maxim violations. Here the Maxim of Relation may play a special role within the domain of ToM reasoning through enjoining listeners to compute and extract relevance in conversations as intended by speakers [48]. Eskritt et al. [23] found that young children were most clearly sensitive to violations of the Relation Maxim and, in our investigation, the bilingual advantage on the CVT shown by German-Italian bilinguals and in our comparison of English-Japanese and Slovenian-Italian bilinguals was clearest for Relation.

Despite the overall pattern of a bilingual advantage, we do not wish to discount cultural variations in the interpretation of specific maxim violations. For example, in our research, children with a Japanese cultural background showed less sensitivity to violations of the Maxim of Quality compared to violations of other maxims. Their responses on Quality are consistent with those of Japanese children on theory of mind false belief tasks. In an extensive study carried out by Naito and Kayama [49], Japanese children have been shown to be considerably delayed until 6 or 7 years of age in their understanding of false beliefs compared to Western children. According to Naito and Kayama, this delay might well reflect the attitudes of Japanese children rather than their ToM competence. In response to unfamiliar “scholar-like” questions, children may strive toward the perceived expectation that an adult questioner would favour a realistic answer that is behaviorally correct. These results are compatible with findings showing that, in Japanese and several other non-Western societies compared to the West, individuals are less likely to be seen as agents acting as autonomous individuals rather than as members of group or community [50]–[52]. Similarly, Japanese children on violations of the Quality Maxim may not so readily identify an untruthful answer as silly or rude if it is seen to be part of pattern designed to fit in with a questioner's expectations.

As Kinzler, Dupoux, and Spelke [53] have observed, early-developing preferences for native-language speakers as friends may serve as a foundation for later-developing preferences and conflicts among social groups. Although the use of more than one language in a culture has often been seen as socially divisive, early bilingualism in the form of native or near native proficiency in two languages as shown in our study may serve to mitigate such conflicts in contributing to an awareness of what it means to communicate effectively with speakers of different languages. Nevertheless, extracting meaning in conversations with others is a complex process [54], [55], and the CVT used in our investigation is not intended to be a fully comprehensive measure of conversational understanding but is restricted to certain key dialogue themes in which children are often involved. Additional research with monolingual and bilingual children is needed toward examining more broadly their sensitivity to other types of maxim violations and its relation to their developing understanding of idioms, irony, metaphors, and sarcasm.
